# An Optimized High Throughput Clean-Up Method Using Mixed-Mode SPE Plate for the Analysis of Free Arachidonic Acid in Plasma by LC-MS/MS

**DOI:** 10.1155/2015/374819

**Published:** 2015-03-19

**Authors:** Wan Wang, Suzi Qin, Linsen Li, Xiaohua Chen, Qunjie Wang, Junfu Wei

**Affiliations:** ^1^School of Material Science and Engineering, Tianjin Polytechnic University, Tianjin 300387, China; ^2^Bonna-Agela Technologies, Tianjin 300462, China; ^3^School of Environment & Chemical Engineering, Tianjin Polytechnic University, Tianjin 300387, China

## Abstract

A high throughput sample preparation method was developed utilizing mixed-mode solid phase extraction (SPE) in 96-well plate format for the determination of free arachidonic acid in plasma by LC-MS/MS. Plasma was mixed with 3% aqueous ammonia and loaded into each well of 96-well plate. After washing with water and methanol sequentially, 3% of formic acid in acetonitrile was used to elute arachidonic acid. The collected fraction was injected onto a reversed phase column at 30°C with mobile phase of acetonitrile/water (70 : 30, v/v) and detected by LC-MS/MS coupled with electrospray ionization (ESI) in multiple reaction monitoring (MRM) mode. The calibration curve ranged from 10 to 2500 ng/mL with sufficient linearity (*r* ^2^ = 0.9999). The recoveries were in the range of 99.38% to 103.21% with RSD less than 6%. The limit of detection is 3 ng/mL.

## 1. Introduction

**Figure 1:**
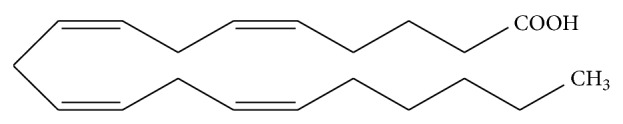
Chemical structure of arachidonic acid.

Arachidonic acid is a *ω*-6 long chain polyunsaturated fatty acid, a senior unsaturated fatty acid. There is high content of free arachidonic acid in the human body which usually comes from dietary animal sources—meat, eggs, and dairy—or is converted from linoleic acid. Arachidonic acid and its metabolites have a strong biological activity and can regulate a wide variety of physiological processes, such as the regulation of lipid and glucose, prevention of cardiovascular disease, chemoprevention of cancer cells, and improvement of memory [1–5]. Therefore, it is important to determine the concentration of free arachidonic acid in human plasma for medical research and clinical diagnosis (Figure 1).

There are many methods to detect arachidonic acid in plasma, such as gas chromatography coupled with mass spectrometry, liquid chromatography with precolumn derivation fluorescence detection, and ELISA method [6–10]. However, these methods are either too complicated to operate or lack of good reproducibility. In contrast, LC-MS/MS has been well accepted for the determination of arachidonic acid with high sensitivity [11–17]. In order to detect arachidonic acid in plasma by LC-MS/MS, the samples have to be pretreated to remove interferences which may result in matrix effect on mass spectrometry [18–21]. Although the traditional techniques such as protein precipitation (PPT), liquid-liquid extraction (LLE), and SPE with reversed phase packing material have been reported for clean-up of plasma samples, it is clear that these techniques are insufficient to remove interferences of phospholipids and proteins in plasma [22–24], while the combination of ionic interaction and reversed phase interaction was reported to remove phospholipids and proteins more sufficiently in the clean-up process for plasma samples [25]. Furthermore, the analytical chemists in the field of preclinical researches and routine clinical testing are facing thousands of samples. Therefore, it is necessary to establish a simple, fast, efficient, and high throughput sample clean-up method for LC-MS/MS analysis of numerous samples [26, 27].

The attention of this study was focused on the development of a high throughput and sufficient sample clean-up method prior to the analysis of arachidonic acid in plasma by LC-MS/MS. Various sample preparation methods including PPT, LLE, single-mode SPE with nonpolar interaction, and mixed-mode SPE with multiple interaction were studied. The matrix effect of both phospholipids and proteins on the recovery of arachidonic acid was investigated and the final method was applied for the assay of some human plasma samples.

## 2. Experimental

### 2.1. Materials and Reagents

Arachidonic acid (purity of 99%), formic acid, and ammonia were purchased from Sigma-Aldrich (St. Louis, MO, USA). Methanol, acetonitrile, and ethyl acetate were of HPLC-grade and were purchased from Merck (Darmstadt, Germany). Purified water was produced by a Milli-Q Academic System (Millipore, Billerica, MA, USA). Human plasma samples were obtained from local hospital.

Cleanert PPT 96-well plate, Cleanert collection 96-well plate (2 mL), Cleanert PEP 96-well plate (60 mg/well), and Cleanert MAS-M 96-well plate (60 mg/well) were purchased from Agela Technologies (Wilmington, Delaware, USA).

### 2.2. Instrumentation

Positive pressure SPE manifold, vortex mixer, and centrifuge for 96-well plate were purchased from Agela Technologies (Wilmington, Delaware, USA).

**Figure 2:**
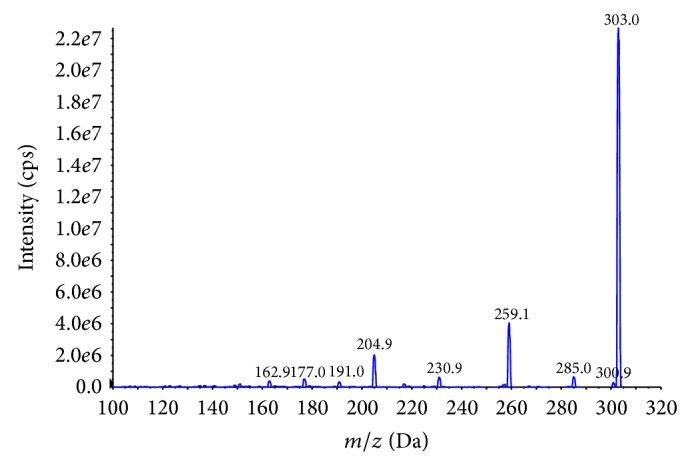
Product ion mass spectra of arachidonic acid.

The analysis was accomplished with a Shimadzu LC-20A binary HPLC system with an autoinjector coupled with API4000+ triple quadrupole tandem mass spectrometer (AB SCIEX, MA, USA). AB SCIEX Analyst software (version 1.5.1) was used for data acquisition. The HPLC column was a 3 *μ*m, 2.1 mm × 50 mm Venusil ASB C18 (Agela Technologies) operated at 30°C under an isocratic condition with mobile phase of acetonitrile/water (75 : 25, v/v). Flow rate was 0.2 mL/min and injection volume was 5 *μ*L. The target compounds eluted from the HPLC column were introduced directly into the MS source. Electrospray ionization (ESI) with negative ion mode was selected for arachidonic acid and that with positive ion mode was selected for phospholipids, respectively. Quantitative analysis was performed under MRM mode by calculating the peak areas. The optimal MS parameters were listed in Table 1. The ions were detected by multiple reaction monitoring (MRM), monitoring the [M + H]^+^ transition of the *m*/*z* precursor ion to the *m*/*z* of the product ion for arachidonic acid. These MS/MS transitions utilized for analysis were *m*/*z* 303/259.1 and 303/205.1. An example of the mass spectra of arachidonic acid was shown in Figure 2. A UV detector at 254 nm wavelength was applied for the detection of proteins.

### 2.3. Standards and Stock Solutions

10 mg of arachidonic acid was dissolved in 100 mL of methanol to prepare a stock solution at 100 *μ*g/mL. The stock solution was further diluted with a mixture of acetonitrile : water (70 : 30, v/v) to obtain work solution with the required concentration of arachidonic acid.

### 2.4. Sample Pretreatments

PPT is a widely adopted sample pretreatment for plasma with routine procedures [22], while LLE and single-mode SPE with reversed phase packing material have been reported to pretreat plasma when arachidonic acid was detected [11, 24]. They were compared with Cleanert MAS-M which was a mixed-mode SPE to extract arachidonic acid in plasma. All the procedures for each sample pretreatment method were described as follows.

5 mL of plasma spiked with 50 *μ*L of arachidonic acid work solution was mixed by vortex for 30 seconds to get homogenate samples.

#### 2.4.1. Method A: Protein Precipitation

100 *μ*L of plasma sample diluted with 100 *μ*L of 1% formic acid was loaded into each well of protein precipitation plate followed by 400 *μ*L of acetonitrile. The plate was vortexed for 30 sec. After centrifuging at 6000 rpm for 5 min, the plate was set on a positive pressure 96-well plate manifold for eluting the target compound into 96-well collection plate. The eluates were dried at 45°C under a gentle stream of nitrogen. The residues were reconstituted with 200 *μ*L of acetonitrile : water (70 : 30, v/v) for LC-MS/MS analysis.

#### 2.4.2. Method B: Liquid-Liquid Extraction

100 *μ*L of plasma sample diluted with 100 *μ*L of 1% formic acid was loaded into each well of 96-well collection plate followed by 5 *μ*L of methanol. After 30 sec vortex, 500 *μ*L of ethyl acetate was added to each well of the plate and then vortexed for 3 min. The plate was stood for 1 min and centrifuged at 6000 rpm for 5 min. The supernatants were transferred into a clean collection plate sequentially and were dried at 45°C under a gentle stream of nitrogen. The residues were reconstituted with 200 *μ*L of acetonitrile : water (70 : 30, v/v) for LC-MS/MS analysis.

#### 2.4.3. Method C: Solid Phase Extraction with Cleanert PEP

100 *μ*L of plasma sample diluted with 100 *μ*L of 1% formic acid was loaded into each well of Cleanert PEP, a single-mode SPE plate packed with reversed phase resin. The plate was preconditioned with 1 mL of methanol and 1 mL of water sequentially. 500 *μ*L of methanol : water (5 : 95, v/v) was used to wash each well of the plate. The target compounds were eluted with 2 mL of 5% ammonia in acetonitrile. The eluates were collected into collection plate and further concentrated at 45°C under a gentle stream of nitrogen until dryness. The residues were reconstituted with 200 *μ*L of acetonitrile : water (70 : 30, v/v) for LC-MS/MS analysis.

#### 2.4.4. Method D: Solid Phase Extraction with Cleanert MAS-M

100 *μ*L of plasma sample diluted with 100 *μ*L of 3% ammonium hydroxide was loaded into each well of Cleanert MAS-M, a mixed-mode SPE plate which was preconditioned with 1 mL of methanol and 1 mL of water sequentially. The plate was washed with 500 *μ*L of water followed by 500 *μ*L of methanol. The target compound was eluted with 600 *μ*L of 3% formic acid in acetonitrile and collected into collection plate. The eluates were concentrated at 45°C under a gentle stream of nitrogen until dryness. The residues were reconstituted with 200 *μ*L of acetonitrile : water (70 : 30, v/v) for LC-MS/MS analysis.

## 3. Results

### 3.1. Comparison of Sample Pretreatment Methods on the Effect of Eliminating Phospholipids

**Figure 3:**
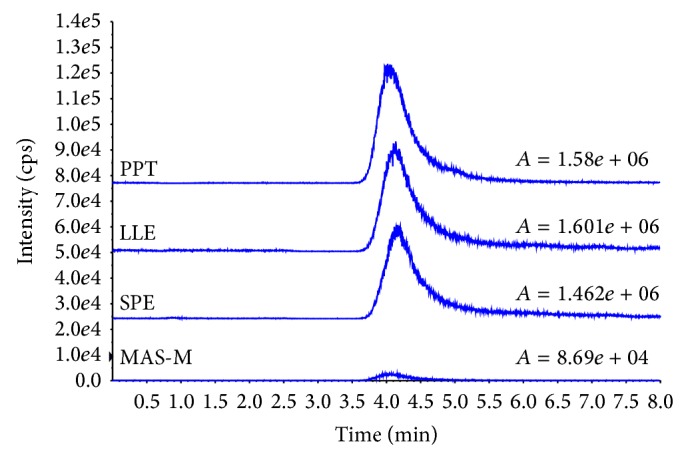
Chromatograms and peak areas of phospholipids in plasma treated with various clean-up methods, where “*A*” = peak area of phospholipids.

It is well known that phospholipids in plasma will result in matrix effect on mass spectrometry [19, 20]. Therefore, it is necessary to remove phospholipids from the samples before injection. Although PPT is a common method for biosample preparation [22], the method is unable to remove the phospholipids from plasma efficiently. As shown in Figure 3 (PPT), the eluate obtained from Method A results in a big phospholipids peak that may influence the analysis of arachidonic acid. Although LLE is a widely used sample preparation method to extract the target compounds from aquatic samples [23], the result of Method B (Figure 3 LLE) indicates that a wide peak of phospholipids appeared. Comparing with LLE, SPE has become a popular sample preparation technique in terms of reproducibility, less usage of organic solvents, and ease of use. Moreover, SPE is very compatible with an automatic system for high throughput analysis. Arachidonic acid is a hydrophobic compound with Log Kow 7.27; in Method C, therefore, a Cleanert PEP plate packed with nonpolar polymer material was used. However, as shown in Figure 3 (SPE), a broad high peak of phospholipids still remains after SPE clean-up. It is apparent to see from Figure 3 (MAS-M) that Method D is the best one for removing phospholipids from plasma. Cleanert MAS-M plate is packed with mixed resins with nonpolar, anion exchange, and cation exchange interactions. Since the pKa of arachidonic acid is 4.77, it is retained on the plate by both anion exchange and nonpolar interactions while the phospholipids and some proteins are retained on the plate by cation exchange and nonpolar interactions under experiment conditions during sample loading. Water soluble interferences are washed out with water and the nonpolar interferences are removed by methanol. Since the arachidonic acid is adsorbed on the plate by anion interaction, there is no loss when the plate is washed by methanol. Finally, after optimizing, 600 *μ*L of 3% formic acid in acetonitrile is applied to release arachidonic acid from the plate while phospholipids with choline groups and proteins with poly amino-groups are retained by the Cleanert MAS plate.

### 3.2. Comparison of Sample Pretreatment Methods on the Effect of Eliminating Proteins

**Figure 4:**
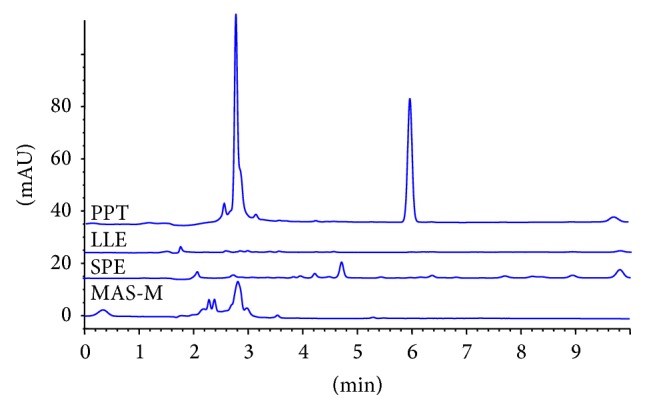
Chromatograms of proteins in samples treated with various clean-up methods, where PPT is Method A, LLE is Method B, SPE is Method C, and MAS-M is Method D.

Proteins with larger molecular weight have similar retention behaviors as arachidonic acid on reversed phase HPLC column. They would cause matrix effect on the determination of arachidonic acid. As shown in Figure 4 (PPT), there are two big peaks that appeared in Method A while the rest of sample pretreatment methods are effective enough to remove proteins from plasma (Figure 4: LLE, SPE, and MAS-M). Combined with the capability for removing phospholipids and proteins, Method D with Cleanert MAS-M plate was selected for further investigation of the analysis of arachidonic acid by LC-MS/MS.

### 3.3. Comparison Study on the Chromatographic Behaviors and Recoveries of Arachidonic Acid of Various Sample Pretreatment Methods

**Figure 5:**
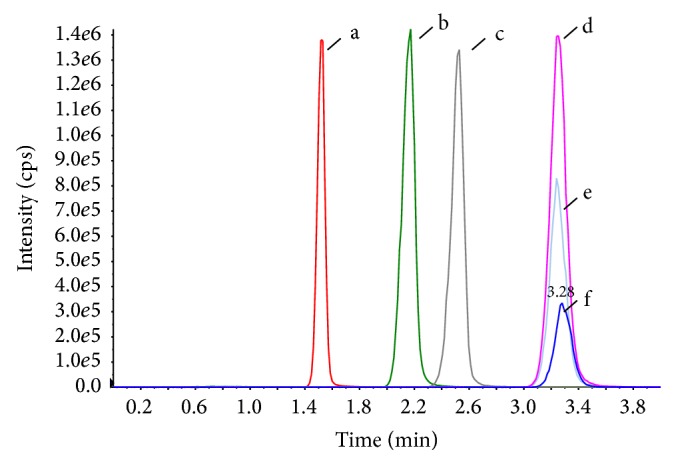
Chromatograms of arachidonic acid in different samples. a: unspiked plasma of Method C, b: unspiked plasma of Method B, c: unspiked sample of Method A, d: spiked plasma of Method D at a concentration of 500 ng/mL, e: unspiked plasma of Method D, and f: arachidonic acid standard, 500 ng/mL.

It is noted that, as shown in Figure 5, the retention time of arachidonic acid in samples treated by Methods A to C is shifted compared with that of arachidonic acid standard. This phenomenon may be due to the accumulation of residual phospholipids and proteins on reversed phase HPLC column. In contrast, the endogenous interferences are removed efficiently in Method D by Cleanert MAS-M; the retention of arachidonic acid is stable. Also, the results listed in Table 2 reveal the advantage of Cleanert MAS-M.

The recoveries of arachidonic acid by various sample pretreatment methods in two concentration levels are listed in Table 2. As discussed in Sections 3.1 and 3.2, the elimination of phospholipids and proteins is not satisfactory by Method A (PPT), Method B (LLE), and Method C (single-mode SPE with Cleanert PEP). The matrix effect caused by phospholipids and proteins is considered as the main factor to cause the variable recoveries of arachidonic acid. In contrast, Method D with Cleanert MAS-M results in efficient removing of impurities and obtains a sufficient, robust recovery of arachidonic acid.

For quantitation, in order to compare the absolute recoveries of various sample preparation methods, the external standard method is used so that the matrix effect on the absolute recoveries can be observed clearly by contrast with Method D and others.

### 3.4. Method Validation

#### 3.4.1. Linearity, Limits of Detection, and Quantitation

The calibration curve range of 10~2500 ng/mL was calculated by a regression analysis of the data to a linear fit with a weighting factor of 1/*x* ^2^ for the ratio of the peak area of arachidonic acid against the nominal concentration. In this range, a linear curve was obtained with correlation coefficients (*r* ^2^) better than 0.9999; the result of the linear regression analysis for arachidonic acid is *y* = 5.18*e* + 3*x* + 9.45*e* + 4 (*r* ^2^ = 0.9999).

The limit of detection, defined as the concentration giving the signal to noise ratio of 3, was estimated to be 3 ng/mL for arachidonic acid.

#### 3.4.2. Precision and Accuracy

The recoveries and precision of the proposed method with Method D are summarized in Table 2. Two concentration levels at 500 ng/mL and 2 *μ*g/mL were measured. The average recoveries are in the range of 99.38%~103.21% with RSD ranged from 5.17% to 5.34%.

It is found that adding 100 *µ*L of 3% ammonium hydroxide to dilute plasma is critical to improve the recovery of arachidonic acid, because it will damage the binding of analyte and proteins in plasma by ionizing arachidonic acid. Also, the ionized arachidonic acid will be adsorbed strongly in the loading process by the resin with the combination of anion exchange and nonpolar interaction. By contrast, the recovery of arachidonic acid was insufficient when 100 *μ*L of water was used to dilute plasma.

### 3.5. Applications of the Proposed Methods

The optimized clean-up method using Cleanert MAS-M plate coupled with LC-MS/MS was applied to analyze free arachidonic acid in human plasma samples obtained from local hospital. The results are listed in Table 3.

The method was also applied in medical research center of local hospital to determine more than 100 actual plasma samples from patients of coronary heart disease and healthy people. With the high throughput sample clean-up method utilizing 96-well plate, the efficiency of the analysis was improved and well accepted by the researchers from local hospital. The results showed the average concentration of free arachidonic acid in the plasma from patients was lower than that from healthy people, which fit the law of pathology.

## 4. Conclusion

An effective clean-up procedure is developed by comparing four different sample pretreatment methods. The selected method (Method D) is simple, accurate, and precise and has high throughput for the determination of arachidonic acid in plasma samples. In contrast to protein precipitation (Method A), liquid-liquid extraction (Method B), and SPE on Cleanert PEP (Method C), Cleanert MAS-M (Method D) method has better effect on eliminating matrix effect of phospholipids and proteins in the analysis of arachidonic acid in plasma by LC-MS/MS. A sufficient recovery with acceptable precision is reached. The proposed method is successfully applied for determining arachidonic acid in human plasma. The results indicate that the method can be used for routine analysis of arachidonic acid in pharmaceutical industries, hospitals, and research laboratories effectively. Since the 96-well plate was used, the clean-up method can easily be automated. This study has shown the possibility to apply Cleanert MAS-M plate for the pretreatment of hydrophobic analytes in plasma which are usually coeluted with phospholipids and proteins on reversed phase HPLC column.

## Figures and Tables

**Table 1:** MS parameters.

Analyte	*t*_*R*_/min	Q1	Q3	DP	CE	IS/V	TEM/°C	GS1/Pa	GS2/Pa	CUR/Pa
Arachidonic acid	3.3	303	259.1	−109	−18	−4500	500	55	35	15
		303	205.1	−107	−20					

Phospholipids	4.1	496.3	184.3	63	20	+5500	600	50	50	15

**Table 2:** Recoveries of arachidonic acid in spiked samples treated by four sample pretreatment methods.

Concentration of arachidonic acid spiked in plasma	PPT (*n* = 5)		LLE (*n* = 5)		SPE (*n* = 5)		MAS-M (*n* = 5)	
	Recoveries (%)	RSD (%)	Recoveries (%)	RSD (%)	Recoveries (%)	RSD (%)	Recoveries (%)	RSD (%)
500 ng/mL	129.32	11.14	85.48	55.90	132.95	19.60	103.21	5.17
2 *μ*g/mL	130.42	2.06	67.05	82.21	94.66	9.21	99.38	5.34

**Table 3:** Free arachidonic acid in some human plasma samples.

Sample number	1	2	3	4	5	6	7	8
Concentration of arachidonic acid (*μ*g/mL)	1.51	0.83	1.15	0.98	1.83	2.11	2.18	1.82
